# An intrusion detection model for electric vehicle supply equipment based on multi-task learning and probability transfer mechanism

**DOI:** 10.1038/s41598-026-42331-3

**Published:** 2026-03-10

**Authors:** Kang Yang, Lizhi Cai, Jianhua Wu

**Affiliations:** 1 Shanghai Key Laboratory of Computer Software Testing & Evaluating, Shanghai, 201112 China; 2 Shanghai Development Center of Computer Software Technology, Shanghai, 201112 China

**Keywords:** Intrusion detection, Electric vehicle supply equipment security, Multi-task learning, Deep learning, Feature representation, Energy science and technology, Engineering, Mathematics and computing

## Abstract

With the rapid development of new energy vehicles and Internet of Things technology, new energy vehicle users have an increasing demand for electric vehicle supply equipment (EVSE). However, a large number of EVSE are currently vulnerable to remote hacking due to the lack of effective security defense mechanisms. This not only leaks sensitive information of users, but also causes large-scale fluctuations in power grid energy. In addition, current research methods for intrusion detection in EVSE suffer from problems such as high false alarm rate, long detection time, and low detection accuracy. Therefore, in order to build a more effective EVSE intrusion detection method, this paper combines the multi-task learning architecture and proposes a deep learning detection model *EVSEMTLIDS* to conduct an in-depth analysis of the malicious intrusion behavior of EVSE. In order to shorten the intrusion detection time of this model for multiple tasks, EVSEMTLIDS integrates a multi-task learning architecture. At the same time, we propose a probability transfer mechanism to improve the prediction performance between multiple tasks. Experimental results show that compared with the baseline model, the proposed model improves the detection accuracy by up to 27.29% and shortens the detection time by 43.24%.

## Introduction

In recent years, with the continuous development of new energy technologies^1,2^, electric vehicles^3^ have accounted for an increasingly high proportion in the transportation industry. Moreover, with the continuous growth of the use rate of electric vehicles^4^, they have also played an important role in new energy and environmental protection^5^. Among them, EVSE^6^ are an indispensable part of current electric vehicles and smart cities^7^, and they are also an important part of the implementation of new energy strategies in various countries. However, when electric vehicles are charged using EVSE, they are connected to the national power grid^8^, so there is a possibility of physical fire after being hacked^9^. In particular, when malicious attackers control the host of the EVSE, they affect the load of the state power grid directly. The simultaneous charging or discharging of the power grid by controlling the power grid not only has a great impact on the stability of the power grid, but also may cause problems such as short circuits in the EVSE^10^, thereby affecting the safety of electric vehicles.

**Fig. 1:**
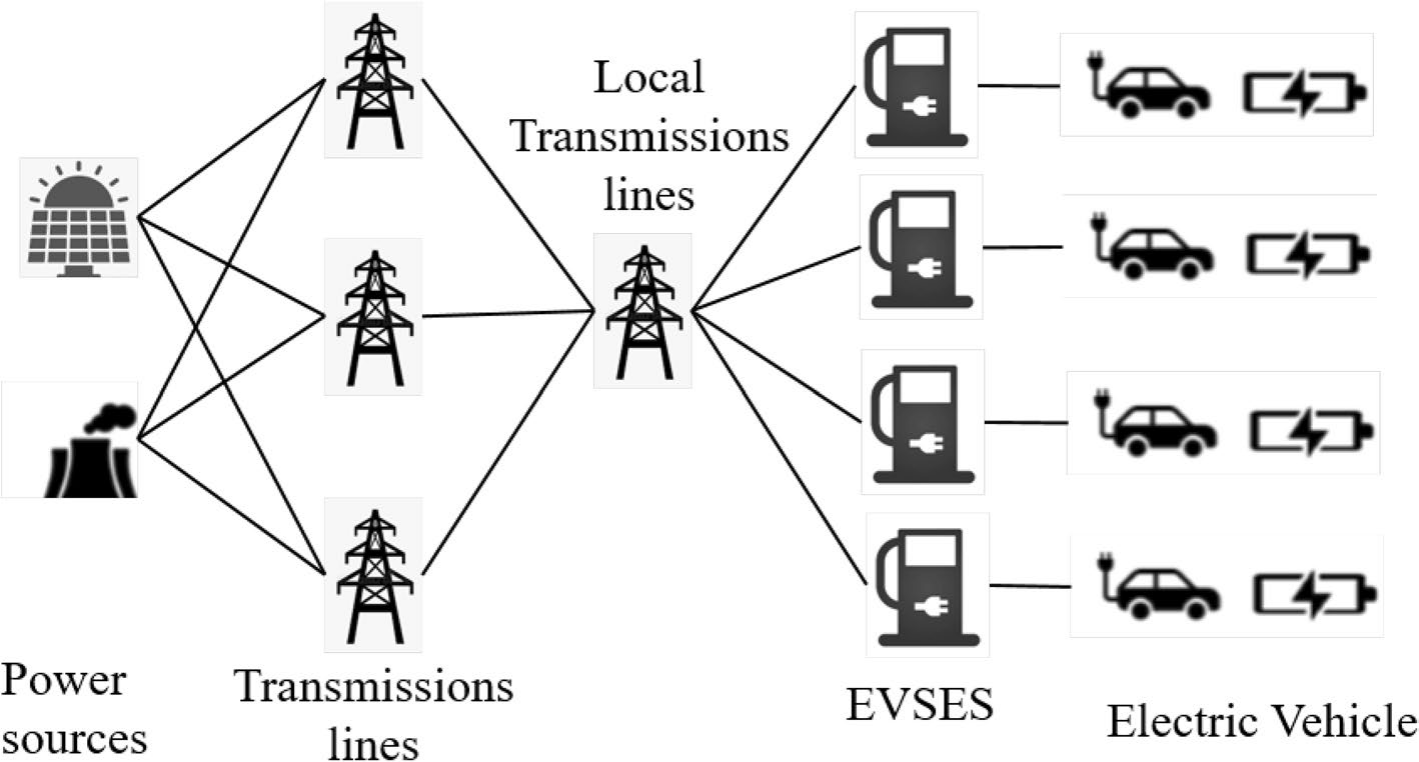
The energy and data flow of EVSE.

With the rapid growth of electric vehicles, the number of electric vehicle EVSE is also increasing. However, a large number of EVSE currently lack effective security defense mechanisms, which makes many EVSE devices to be hacked. For example, the intrusion security issues have EVSE DoS attack and backdoor vulnerabilities, etc. These malicious intrusions only increases the risk of user privacy information leakage, but also raises the threat to national energy security. In the past years, cyber attacks on the IT systems of power grid operators have been caused serious power grid blackouts^11^. These intrusions pose a serious challenge to the safety of EVSE, and the current EVSE intrusion detection work still has some common problems:

Firstly, in the current EVSE intrusion detection work, the network flow features of EVSE are widely used. For example, researchers use flow characteristics to detect malicious behavior. However, these flow data have high overlap rate, low detection model recognition, and high false alarm rate. For example, the IP address and port in the flow characteristics are basically the same in each sample data, and their feature distribution is similar, which makes it difficult to use them for model detection. To solve this problem, Fu et al.^12^ tried to convert these flow data into frequency characteristics, but this method cannot completely solve the problem of high overlap of flow characteristics.

Secondly, the current EVSE detection methods can only detect whether they are malicious or benign, but cannot analyze their inherent malicious behavior. Researchers can only classify the intrusion traffic in the EVSE as malicious, that is, predict the data label as 0 or 1. Although these works can effectively predict the category of samples, they cannot deeply explore the malicious behavior of these samples, and it is difficult to build a security defense mechanism in time to reduce the loss of the hacked device.

Finally, if the current EVSE detection method need detect the fine-grained malicious behavior of the sample, it is necessary to build new detection model. This situation results in longer model training time. Besides, the repeated sample characterization process prolongs the detection time of the model and delays the generation of the model prediction results.

Therefore, to solve the above problems, we constructs an effective EVSE intrusion detection system based on host features. This paper proposes a detection architecture EVSEMTLIDS that combines multi-task and deep learning. The EVSEMTLIDS model can effectively reduce the detection time of the training and improve the prediction performance between each task of the model.

Unlike existing MTL techniques such as PLE, Gated Transfer Networks, or Task-wise Attention, which primarily focus on latent feature-level decoupling to mitigate negative transfer, our proposed Probability Transfer Mechanism (PTM) introduces an explicit logical constraint across the task hierarchy. While conventional methods recalibrate high-dimensional feature weights, PTM operates in the low-dimensional probability space by mapping posterior distributions from upstream binary detection to downstream fine-grained classification via a transformation matrix $W_p$. This probabilistic prior ensures semantic consistency across tasks, where fine-grained predictions are logically bounded by coarse-grained confidence—effectively suppressing error propagation and reducing false alarms in a way that feature-based gating or attention mechanisms cannot.

The main contributions of this paper are divided into the following points.

- This paper constructs EVSEMTLIDS detection model that deeply integrates host and network features, effectively overcoming the high overlap of traditional traffic features.
- The model utilizes a multi-task collaborative architecture to achieve fine-grained classification of malicious behavior, breaking through the limitations of binary classification and accurately identifying attack subtypes.
- EVSEMTLIDS employs a multi-task parallel computing framework, simultaneously generating multi-task representations within a single forward propagation, significantly reducing detection latency.
- The model innovatively introduces a probability transfer mechanism to optimize multi-task correlation, addressing the negative transfer problem of traditional methods and demonstrating superior performance in key metrics.

The rest of the paper is organized as follows. “Related work” provides a detailed overview of the relevant background, including EVSE intrusion detection and Multi-task learning method. In “Proposed method”, we describe the EVSEMTLIDS detection architecture in detail, including data preprocessing, data representation, and Probability transfer mechanism. “Experiment set up” provides an extensive discussion of the dataset and experimental setup for the experiments, and explains the metrics for the performance of the EVSE intrusion detection method. “Evaluation and discussion” validates the effectiveness of the EVSEMTLIDS model through relevant experiments in the context of five research questions. In “Conclusion and future works”, the conclusion of this paper and research directions for future work are summarized.

## Related work

The related work can be divided into the following three main parts: (1) current challenges, (2) EVSE intrusion detection; (3) multi-task learning method.

**Fig. 2:**
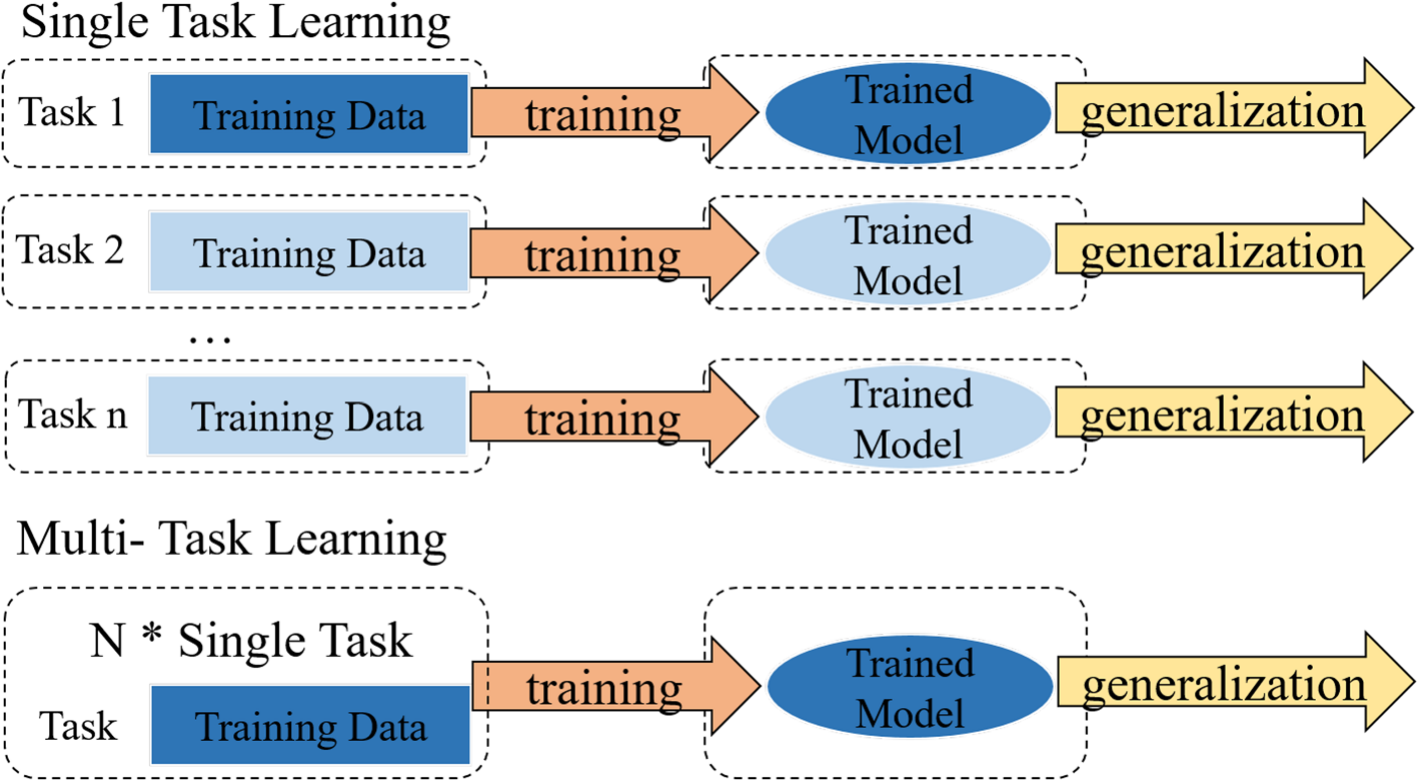
The multi-task learning method.

### Current challenges

EVSE is facing an increasingly severe threat from cyberattacks. These attacks not only threaten user privacy and financial security but also pose a serious threat to the stable operation of the power grid. Figure 1 illustrates the current power grid transmission process, showing the energy and data transmission flow between the National Energy Administration, the State Grid, EVSEs, and electric vehicles. National energy includes various energy sources, such as solar and wind power. This energy is converted into electricity and then transmitted to the energy grid in each city via the State Grid’s transmission lines. Subsequently, energy managers in each city, in conjunction with the management system, wirelessly dispatch energy to the EVSEs in each region. Finally, each electric vehicle user charges their electric vehicle using the EVSE device by scanning a QR code or logging into a client to send a charging request to the management system. In February 2022, Notable attacks in Russia^13^ involved Denial of Service (DoS) disruptions to EVSE operations.

Currently, false data injection^14,15^ directly target power grid dispatch systems. Attackers tamper with charging demand data, misleading the grid dispatch system and causing local distribution network overloads, resulting in line tripping and threatening regional power supply stability. However, as a complex cyber-physical system, the electric vehicle power supply system is vulnerable to a wider range of attacks, including communication networks, charging equipment, and the vehicle itself. Attackers often exploit protocol vulnerabilities, system defects, and physical interfaces to launch attacks. EVSEs are also subject to DDos attacks^16^, which can cause service interruptions and resource exhaustion, making them unable to respond to legitimate users’ charging needs. Man-in-the-middle attacks^17^ also affect EVSEs, allowing attackers to intercept and tamper with communication data between electric vehicles and charging stations, stealing sensitive privacy such as user account credentials, and directly causing financial losses to users. EVSEs have also been subject to load tampering network attacks^18^. These issues can allow attackers to manipulate the coordinated charging and discharging of a large number of charging stations, causing abnormal grid frequency and voltage instability.

To improve the detection accuracy of EVSE intrusion events, researchers have explored the use of machine learning and deep learning technologies. However, these current detection methods struggle to address the high overlap of network traffic features. Furthermore, these intrusion detection models consume a long time for multi-class detection. A multi-task learning architecture can share model data representations, thereby shortening detection time. The overall architecture of multi-task is shown in Figure 2. Multi-task learning can share features with each task, which has better feature representation than the single-task model.

### EVSE intrusion detection

EVSE intrusion detection is an important part of the security of energy power grids, and it also plays a key role in the driving safety of new energy electric vehicles. Traditional IoT device intrusions^19,20^ will invade the IoT to steal relevant user privacy data^21^. Studies have shown that the purpose of these intrusions is not to destroy related physical devices but to extract data assets, so the intrusion harm is relatively small. EVSE is an important part of power grids infrastructure. Once invaded, they may cause damage to physical equipment, even cause electric vehicle batteries to catch fire^9^, which is endanger human safety. In addition, EVSE intrusions will also lead to large-scale charging and discharging, it will bring a greater burden to the power grid. Therefore, EVSE intrusions are more harmful than traditional IoT intrusions and have a wider range of harm. At present, there have been some related studies on EVSE security.

In order to solve the problem of EVSE being hacked and refusing to charge, or damaging the battery pack due to overcharging, dey et al^17^ designed a model for detecting cyber attacks that may affect PEV battery packs^22^ during the charging process. The model is designed as a filter-based dynamic detector, taking into account multi-target detection criteria including stability, robustness, and attack sensitivity. However, the detection accuracy of traditional methods is limited. To solve this problem, researchers used machine learning methods to detect intrusions. Among them, in order to avoid the impact of EVSE on the local power dispatching network after being invaded, Chung et al.^23^ using the multivariate time series segmentation^24^ method and the KNN classifier^25^ to perform intrusion detection analysis on EVSE and power grid dispatching data, thereby identifying abnormal data in the power network. In order to solve the problem that the traditional threshold method cannot detect abnormal points or faults in the charging process of electric vehicles, researchers^26^ proposed a charging anomaly detection method based on a multivariate Gaussian distribution^27^ model. This method first preprocesses the electric vehicle charging data and obtains the anomaly detection categories and standard curves through data visualization. Researchers proposed a classifier-based billing session collaborative anomaly detection system for EVSE session traffic data^28^, focusing on the coordination between different IDS instances and simulating attacks through random anomalies. In order to solve the problems of high false alarm rate in traditional machine learning algorithms, Cumplido et al.^29^ proposed a collaborative anomaly detection system CADS4CS for charging stations as an optimization measure. The CADS4CS model is a solution that can accurately detect unforeseen events and possible fraud threats that occur during the charging process in charging stations, which effectively improving the detection efficiency of the model.

However, most of the above detection models are based on network traffic features, and the overlap between abnormal and benign data is higher, which directly leads to a high false alarm rate of the detection model. In this work, we study the host features of the EVSE to detect whether the EVSE is hacked. In addition, in order to reduce the detection time, we use a multi-task learning method to uniformly represent the data for the samples.

### Multi-task learning method

Multi-task learning architectures can accelerate model learning by leveraging shared features between related tasks, and reduce the computational time of the testing process^30^. Specifically, a multi-task learning framework that includes a set of tasks can provide more accurate output results than a single-task framework.

At present, multi-task learning is involved in many fields, such as face recognition^31,32^ , emotion prediction^33^, text classification^34^ and other popular fields. Inspired by these studies, researchers found that multi-task learning can also be applied to intrusion detection. After analyzing the network traffic characteristics visually, the researchers^35^ proposed to mine the unique characteristics of each type of traffic from three perspectives: anomaly identification, clustering, and classification. And combined with a multi-task deep learning architecture to detect these three types of tasks. The researchers believe that most existing methods usually only utilize a specific aspect of network traffic features and regard model training as a single-task learning problem, thereby ignoring the discriminative power of different feature types and the performance enhancement of integrating multiple machine learning tasks. Therefore, they proposed a multi-task learning model MEMBER^36^ with mixed deep features, and introduced two auxiliary tasks, namely an autoencoder with a memory module and a distance-based prototype network, to improve the model generalization ability and alleviate performance degradation in unbalanced network environments. In addition to traditional deep learning models, researchers^37^ proposed a federated learning multi-task deep neural network to simultaneously perform tasks such as network anomaly detection and traffic classification. Compared with multiple single-task detection models, this method can effectively reduce training time overhead.

However, there is a problem of mutual influence between the above detection tasks^38^, that is, the tasks cannot reach the optimal value at the same time, which is due to the conflict between the optimal solutions of the tasks. In order to solve this problem, we proposed a probability transfer mechanism, which can effectively transfer the probabilities between tasks, thereby constructing a reasonable loss function and improving the prediction performance of the model.

## Proposed method

### Overview

**Fig. 3:**
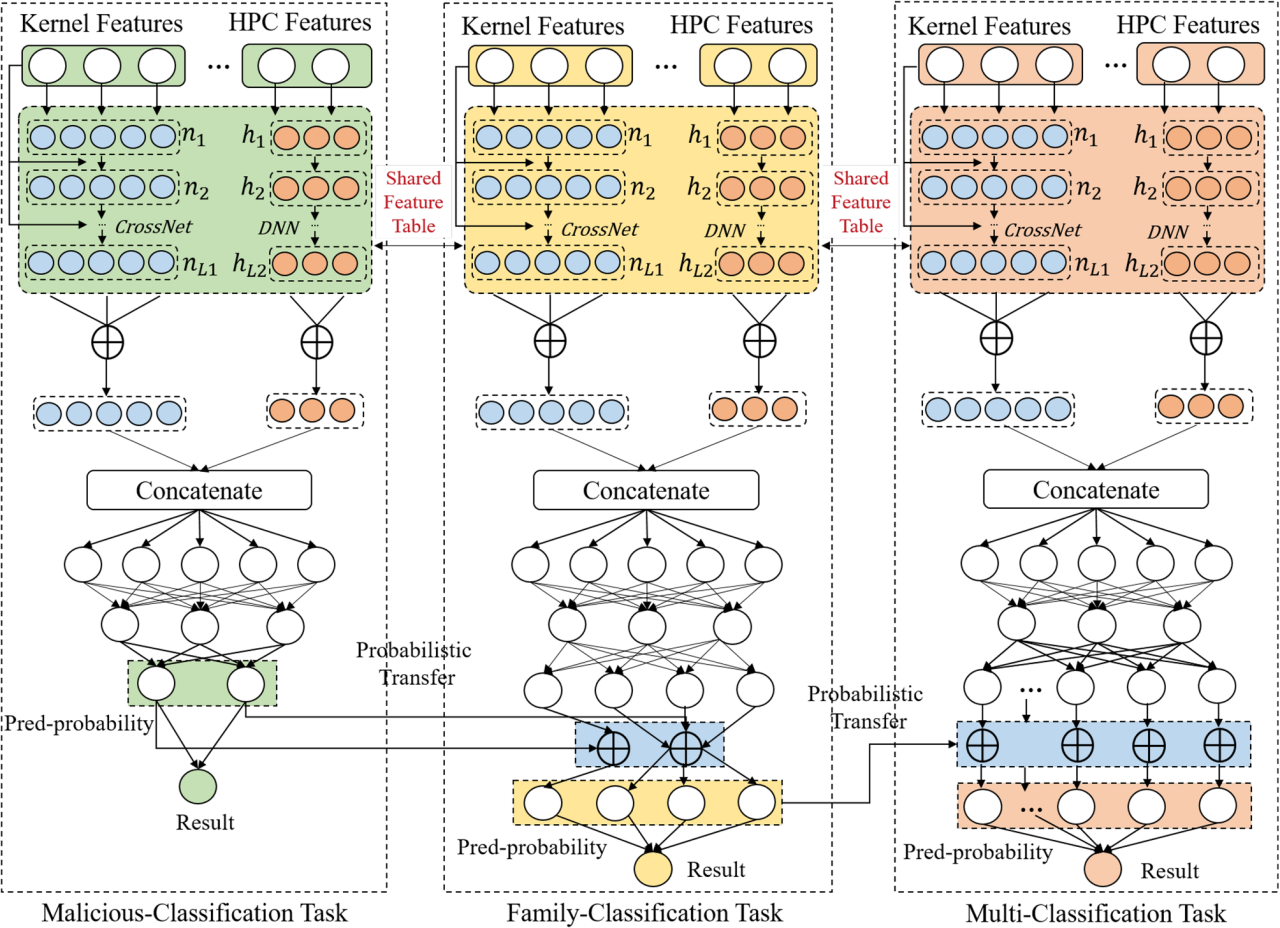
The overall architecture of the EVSEMTLIDS model.

In this section, we describe the EVSEMTLIDS method in detail. First, we introduce the preprocessing part of the sample data and divide the data features into sparse and dense features. Subsequently, the neural network method was used to quantitatively characterize the sample data. Finally, the multi-task learning architecture and probability transfer mechanism are combined to predict the samples. An overall overview of the EVSEMTLIDS method is shown in Fig. 3.

### Data preprocessing

Data preprocessing is the process of normalizing feature data. First, we delete all invalid features in the data set. The sample data of these features are consistent, that is, the data of this feature are the same in each sample. For example, in the host feature, all are 0 in the experimental data set. These features not only fail to effectively guide the model to make effective predictions, but also increase the computational complexity of the model. Therefore, we filter and remove these invalid features.

Subsequently, considering that the data distribution of different features is inconsistent, using the same feature representation method will reduce the detection accuracy of the model. Therefore, the model divides features into sparse and dense. We count the proportion parameter *a* of sample 0 in each feature, and the calculation is as shown in Eq. (1):

1 $$\begin{aligned} a = sum(x_{i}=0 )/N \end{aligned}$$

The *sum*() function is the number of samples that are 0 in the statistical feature $x_i$, and *N* is the total number of samples. Then, we introduce the threshold parameter *b* and use the size of parameter *a* to classify the features. The sparse and dense features are divided as shown in Eq. (2):

2 $$\begin{aligned} x_{i}={\left\{ \begin{array}{ll} & x_{s} \quad \text { if } a> b \\ & x_{d} \quad \text { if } a\le b \end{array}\right. } \end{aligned}$$

For any feature $x_i$, if its calculated parameter *a* is greater than the threshold parameter *b*, it is classified as sparse. Otherwise, the feature $x_i$ is divided into dense features. In addition, in order to avoid gradient explosion during detection model training and improve the detection effect of the model. We normalize the dense features and use the z-score feature standardization method to unify the sample feature distribution. The calculation method is as shown in Eq. (3):

3 $$\begin{aligned} x_{d} = \frac{x_{d}-\mu _{d} }{\delta _{d}} \end{aligned}$$

Among them, $x_d$ is the dense feature of any one of the samples *x*, $\mu _{d}$ and $\delta _{d}$ are the mean and variance of the feature data respectively. Existing research shows that the z-score method is better than the traditional normalization operation in multi feature dimensionality reduction.

In addition, since sparse features contain less data information, normalization processing is difficult to improve the representation effect of feature data. For this reason, we use dictionary mapping method to characterize samples, and the transformation process is shown in Eq. (4):

4 $$\begin{aligned} x_{s} = embed\_dic(x_{s}) \end{aligned}$$

Finally, we splice the discrete data of dense and the sparse feature data. The $x_0$ is the final sample feature data, as shown in Eq. (5):

5 $$\begin{aligned} x_{0} = [x_{d},x_{s}] \end{aligned}$$

### Data representation

In order to effectively characterize the characteristics of the sample, we use two deep neural networks, DNN and CrossNet to quantitatively represent all features of the sample data. DNN can capture nonlinear high-order features, and CrossNet can capture high-order intersections between features. This network structure combination can automatically learn feature intersections for sparse and dense feature inputs, and it also can effectively capture feature intersections on bounded degrees without manual feature engineering or exhaustive searching, and has low computational cost.

Therefore, the representation of vectors in our multi-task learning architecture is a joint representation of two deep networks, divided into DNN and CrossNet networks. The vector representation of the DNN deep learning model is shown in Eq. (6):

6 $$\begin{aligned} h_1=Relu(W_{h,0}* x_0+b_{h,0}) \end{aligned}$$

Among them, $x_0$ is the initial feature representation of the sample, $h_1$ is the vector representation after DNN deep neural network learning, $W_{h,0}$ is the initial transformation matrix, $b_{h,0}$ is the initial bias value, and *Relu* is the activation function.

After $l_1$ rounds of iterations, the vector representation of the DNN deep learning model is as shown in Eq. (7):

7 $$\begin{aligned} h_{1_{1}} =Relu(W_{h,1_{1}-1}* h_{{1}-1}+b_{h,{1}-1}) \end{aligned}$$

Among them, $h_{1_{1}}$ is the vector representation after iteration of $l_1$ rounds of the sample, $h_{1_{1}}$ is the vector representation after $l_1-1$ rounds, $W_{h,1_{1}-1}$ is the transformation matrix, $b_{h,{1}-1}$ is the offset value.

The vector representation of the CrossNet network is shown in Eq. (8):

8 $$\begin{aligned} n_1=x_0 x_0^T W_{c,0}+x_0 \end{aligned}$$

Among them, $x_0$ is the initial representation of the sample, $n_1$ is the vector representation after deep learning, $W_{c,0}$ is the transformation matrix, and $b_{c,0}$ is the offset value.

After $l_2$ rounds of iteration, the vector representation of the model is shown in Eq. (9):

9 $$\begin{aligned} n_{l_2}=x_0 n_{l_2-1}^T W_{c,l_2-1}+n_{l_2-1} \end{aligned}$$

Among them, $n_{l_2-1}$ is the vector representation of the iteration after $l_2-1$ rounds of the sample, $n_{l_2}$ is the vector representation after deep learning, and $W_{c,l_2-1}$ is the transformation matrix of the model.

Subsequently, the model splices the vectors of the DNN and CrossNet deep learning networks to obtain the sample representation of multi-task learning. The splicing result of the vectors is shown in Eq. (10):

10 $$\begin{aligned} x_{emb}=W_{s,l} [h_{l_1},n_{l_2}] \end{aligned}$$

Among them, $x_{emb}$ is the final vector representation of the sample, $h_{l_1}$ is the vector representation after deep learning, $n_{l_2}$ is the vector representation after deep learning, and $W_{s,l}$ is the transformation matrix.

Finally, the model uses the sigmoid activation function to convert the feature vector of the model into probability output. The calculation process is as shown in Eq. (11):

11 $$\begin{aligned} p=sigmoid(W_p x_{emb}+b_p) \end{aligned}$$

Since the multi-task model assigns the same vector representation to each task, the probability p of different tasks is considered to have different output dimensions. Therefore, combining Eq. (11), the predicted probability vector $p_{bin}$ of malicious detection in Task 1, the predicted probability $p_{fam}$ in Task 2, and the predicted probability $p_{mtl}$ in task three can be generated.

The architectural choice of DNN combined with CrossNet is strategically tailored for the tabular and heterogeneous nature of EVSE host-level data. While Transformers are powerful for sequential data, their quadratic complexity and lack of inductive bias for tabular feature interactions make them less efficient for high-dimensional performance counters. Similarly, Graph Neural Networks (GNNs), despite their ability to model process relationships, introduce significant computational burdens on resource-constrained embedded controllers due to real-time graph construction. The DNN + CrossNet architecture ensures that the intrusion detection system can identify complex attack patterns by cross-referencing multi-dimensional host features without exhausting the limited CPU/RAM resources of the charging station.

### Probability transfer mechanism and model prediction

**Figure 4:**
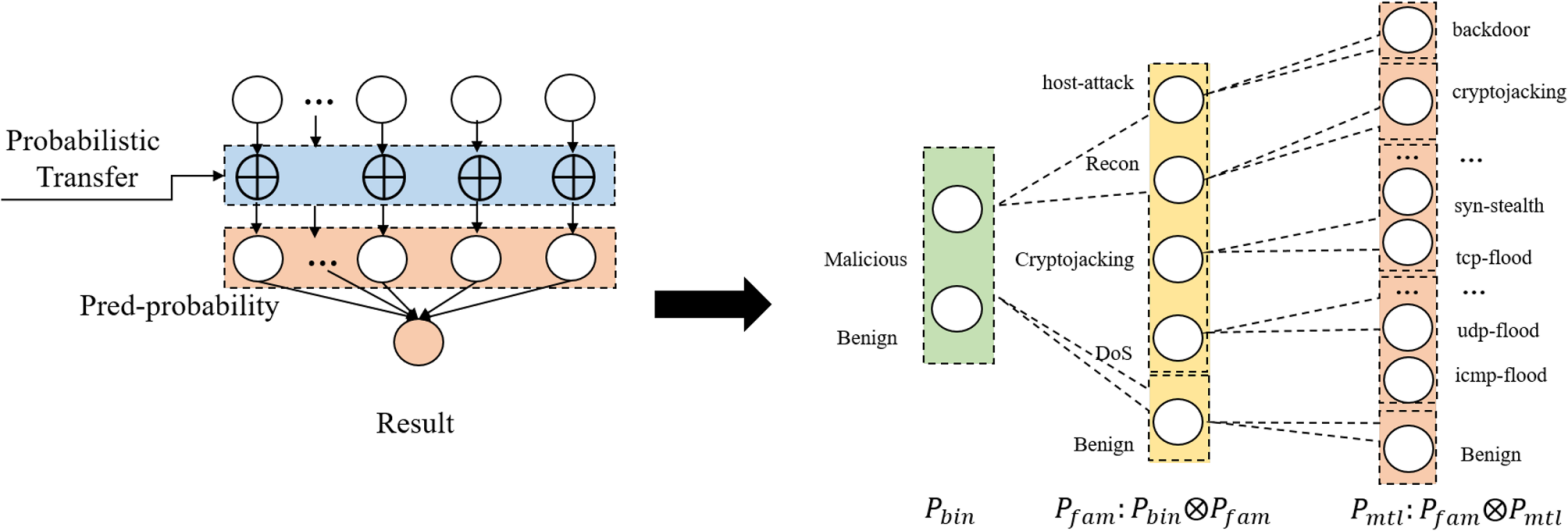
Probability transfer mechanism.

After obtaining the preliminary predicted probability of each task, we consider the correlation of feature information between each task. Therefore, this model proposes a probability transfer mechanism to update the probability output of multi-tasks. The probability transformation structure of the model and the calculation form of the probability transformation is shown in Fig. 4:

First, the label calculation of the model for malicious intrusion detection in Task 1 is shown in Eq. (12):

12 $$\begin{aligned} y=argmax(p_{bin}) \end{aligned}$$

Subsequently, we transformed the output probabilities in Task 1 into the probabilities of the five family classifications. Through probability transfer calculation, the prediction result of family intrusion detection classification in task 2 is shown in Eq. (13):

13 $$\begin{aligned} p_{fam}=\sigma (W_p*p_{bin}) \otimes p_{fam} \end{aligned}$$

$W_p$ is the transformation matrix, $\sigma$ is the activation function, and $\otimes$ is the element wise product. Through the above symbolic operations, the prediction probability $p_{fam}$ can be updated, and the final family prediction label of the sample is shown in Eq. (14):

14 $$\begin{aligned} k=argmax(p_{fam}) \end{aligned}$$

Through probability transfer calculation, the prediction result of multi-classification intrusion detection for task three is shown in Eq. (15):

15 $$\begin{aligned} p_{mtl}=\sigma (W_p*p_{fam})\otimes p_{mtl} \end{aligned}$$

Among them, $p_{fam}$ is the updated classification probability value in Task 2, and $p_{mtl}$ is the probability value of the multi-classification task. The transformation matrix $W_p$ is a parameterized mapping learned during the offline training stage. Specifically, it is integrated into the joint optimization process, where its weights are updated via backpropagation to minimize the global loss across all hierarchical tasks. Unlike dynamically generated attention weights, a learned $W_p$ captures the inherent semantic correlations between different label spaces based on the entire training distribution. This approach not only ensures that the transferred probabilistic prior is grounded in empirical task logic but also maintains inference efficiency, as the fixed matrix multiplication requires minimal computational resources during real-time execution on EVSE hardware. The label of the final multi-class prediction task of the sample is shown in Eq. (16):

16 $$\begin{aligned} z=argmax(p_{mtl}) \end{aligned}$$

Finally, the detection model calculates the loss value between the predicted labels *y*, *k*, *z* and the real value, and continuously optimizes the model parameters through backpropagation of deep learning. In addition, this model uses a combination of multi-task loss functions as the loss function of the model for training. The loss function is as shown in Eq. (17):

17 $$\begin{aligned} L(\theta _{bin},\theta _{fam},\theta _{mtl},x_i )=\lambda _1 loss(x_i,\theta _{bin} )+\lambda _2 loss(x_i,\theta _{fam}) + \lambda _3 loss(x_i,\theta _{mtl}) \end{aligned}$$

Among them, $\theta _{bin}$,$\theta _{fam}$,$\theta _{mtl}$ are the parameters of the loss function of each task, $x_i$ is the sample. The hyper-parameters $(\lambda _1, \lambda _2, \lambda _3)$ controlling the task-wise contribution to the joint loss function were determined through empirical validation and grid search. Specifically, we adopted a hierarchical weighting strategy where the binary detection task is assigned a higher priority to establish a robust foundational prior for the Probability Transfer Mechanism. This fixed-weight approach was selected over dynamic methods like GradNorm to ensure training stability and to explicitly reflect the logical importance of upstream tasks in the progressive detection pipeline. Experimental results on the validation set confirmed that this configuration effectively balances the convergence rates across different classification granularities without the computational instability often associated with dynamic gradient balancing.

Among them, the malicious classification loss function of task 1 is shown in Eq. (18):

18 $$\begin{aligned} L(x_i,\theta _{bin} )=\sum _{i=1}^{N} l(y_i,p_{bin} (x_i;\theta _{bin})) \end{aligned}$$

Among them, the family classification loss function of task 2 is shown in Eq. (19):

19 $$\begin{aligned} L(x_i,\theta _{fam} )=\sum _{i=1}^{N} l(y_i,k_i,p_{bin} (x_i;\theta _{bin}),p_{fam} (x_i;\theta _{fam})) \end{aligned}$$

Among them, the multi-classification loss function of task three is shown in Eq. (20):

20 $$\begin{aligned} L(x_i,\theta _{mtl} )=\sum _{i=1}^{N} l(y_i,k_i,z_i,p_{bin} (x_i;\theta _{bin}),p_{fam} (x_i;\theta _{fam}), p_{mtl} (x_i;\theta _{mtl})) \end{aligned}$$

The parameters $p_{bin} (x_i;\theta _{bin})$, $p_{fam} (x_i;\theta _{fam})$, and the $p_{mtl} (x_i;\theta _{mtl})$ in the above Eqs. (18), (19), and (20) are the predicted probabilities of the three detection tasks under the influence of different loss functions value.

The proposed EVSEMTLIDS architecture incorporates a hierarchical design that effectively mitigates the risk of error propagation through two synergistic mechanisms. First, the PTM replaces conventional “hard” discrete cascading with “soft” probabilistic guidance. By transferring the entire posterior probability distribution rather than a binary label, PTM allows downstream tasks to treat upstream outputs as a probabilistic prior bias. This ensures that subsequent tasks, supported by their specific feature streams (HPC and kernel events), retain the analytical capacity to self-correct even when upstream confidence is suboptimal. Second, the model employs a joint loss function with learnable aggregation parameters, enabling simultaneous optimization of all hierarchical tasks. This global training strategy prevents downstream tasks from blindly replicating upstream classification errors; instead, it enforces a balanced weight between the transferred prior and the local task evidence.

## Experiment set up

### Research questions

In order to verify the effectiveness of the EVSEMTLIDS model, this section proposes the following four experimental questions from the aspects of the overall experimental performance of the model, effects of different parameter settings, ablation experiments of model composition, and model prediction cost.

RQ1: How does the classification effect of the EVSEMTL IDS model compare with the baseline model?

RQ2: How does the main model performance results of the EVSEMTLIDS model?

RQ3: How do the main model parameters affect the experimental results of the EVSEMTLIDS model?

RQ4: How does the ablation experimental effect of the main components in the EVSEMTLIDS model perform?

RQ5: How does the EVSEMTLIDS model compare to the baseline model in terms of time consumption?

RQ6: How does the EVSEMTLIDS model perform in terms of generalization on other datasets compared with the baseline models?

### Dataset and task classification

In order to verify the effectiveness of the EVSEMTLIDS model, experimental validation was conducted using the real public EVSE host intrusion dataset CICEVSE2024. This dataset was released by the Canadian Institute for Cybersecurity in March 2024, which includes 2302 normal samples and 6166 abnormal samples. In addition, the dataset includes three types of labels, which can be divided into two-class detection of malicious intrusion, five-class detection of family intrusion, and multi-classification twenty-class intrusion detection. The statistical information of the CICEVSE2024 dataset label is shown in Tables 1 and 2. Therefore, we can divide the dataset into three detection tasks and use the EVSEMTLIDS model for multi-task learning prediction.

**Table 1:** Dataset label classification.

Malicious	Family	Category			Total
Benign	Benign	Benign			1
Malicious	Cryptojacking	Cryptojacking			1
	Recon	Aggressive-scan	Os-fingerprinting	Port-scan	9
		Serice-detection	Syn-stealth	Vuln-sca	
		Service-detection	Tcp-flood	Os-scan	
	Dos	Icmp-flood	Icmp-fragmentation_old	Push-ack-flood	8
		Syn-flood	Tcp-flood	Udp-flood	
		Icmp-fragmentation	Synonymous-ip-flood		
	Host-attack	Backdoor			1
Task1:Bin-Class	Task2:Fam-Class	Task3:Mtl-Class			Multi-task-class

**Table 2:** CICEVSE2024 dataset statistics.

Bin-class		Fam-class		mtl-class	
Category	Total	Category	Total	Category	Total
Number	8468	Cryptojacking	1793	backdoor	2302
Malicious	6166	Dos	865	Cryptojacking	1793
Benign	2302	Host-attack	2302	Port-scan	201
		Recon	1206	Vuln-scan	192

In addition, the host features of the CICEVSE2024 dataset are mainly divided into two categories, HPC and kernel features. HPC event features mainly include host execution instructions and data cache access and reading behavior features, while kernel features mainly include host memory event behaviors and device transmission data features. Table 3 shows some host feature explanations.

The practical feasibility of acquiring host-level features is substantiated by the CICEVSE2024 dataset, which was collected using standard Linux kernel utilities (e.g., perf) on resource-constrained embedded controllers. In real-world distributed EVSE deployments, this means host features can be monitored without hardware modifications. Regarding computational overhead, our approach employs selective feature extraction—focusing only on the most significant HPC and kernel metrics identified in CICEVSE2024—ensuring that CPU and memory utilization remain minimal (typically below 5%). This edge-based monitoring strategy allows for the real-time detection of stealthy threats (e.g., cryptojacking or backdoors) that are invisible to network-only IDS, while avoiding the latency and bandwidth costs associated with centralized data processing.

**Table 3:** Part of HPC and kernel features.

Category	Feature	Description
HPC	Instructions	Execute instructions
	Cache_misse	Cache misses
	Exc_Taken	Execute action
Kernel	kmem kfree	Memory release
	Net_dev_xmit	device transmission
	Raw_syscall_sys_enter	Raw system read

### Evaluation metric

Since the accuracy metric cannot fully reflect the performance of the detection model in detection tasks, we comprehensively consider five metric including the F1 value. The four automated metric are accuracy, precision, recall and F1 value. The calculation equations for these metrics are as follows:

21 $$\begin{aligned} Accuracy = \frac{TP+TN}{FP+FN+TP+TN} \end{aligned}$$

22 $$\begin{aligned} Precision = \frac{TP}{FP+TP} \end{aligned}$$

23 $$\begin{aligned} Recall = \frac{TP}{FN+TP} \end{aligned}$$

24 $$\begin{aligned} F1 = 2* \frac{Precision \times Recall}{Precision+Recall} \end{aligned}$$

In addition, we consider the time cost metric for training the model in the experimental section.

### Baselines

Since previous studies have been applied to baseline datasets, we need to compare the EVSEMTLIDS model with related baseline models to analyze the performance of our proposed method. We selected eleven intrusion detection models for comparison, and these baseline models can be divided into three categories: (1) detection models based on classic machine learning. (2) Detection model based on homogeneous graph node representation. (3) Detection model based on heterogeneous representation of nodes in the information network.

DNN^39^: DNN model is a kind of deep neural network. Its training process relies on propagation algorithm and gradient descent algorithm. It calculates the error loss value between the output layer and the true label, and propagates the error back to each neuron, updates the weight and bias of the neuron, so as to improve the model’s ability to fit the data.

BPNN^40^: BPNN model is similar to DNN network architecture, but it can achieve better data feature extraction effect by appropriately increasing the number of hidden layers, and is more suitable for processing simpler data, such as text, numerical values, etc.

LSTM-Attention^41^: This model is a detection model that combines LSTM with Attention mechanism. Compared with DNN model, LSTM has stronger memory effect and can effectively deal with the problems of long-term information preservation and short-term input missing in hidden variable model. And combined with attention mechanism, it can effectively improve the weight of important features of the model.

DT^42^: This model is a tree data structure that displays decision rules and classification results. It usually includes three parts: feature selection, decision tree generation, and decision tree pruning.

K-nearest neighbors (KNN)^43^ : The KNN model can predict samples through supervised learning methods. Its detection principle is to classify the target sample based on the majority of the k most similar samples in the feature space.

Naive Bayes^44^ : The NB model is a classification method based on the Bayesian theorem and the assumption of independence of feature conditions. It is a generative model of supervised learning.

Adaboost^45^ : Adaboost is an iterative algorithm. Its core idea is to train different weak classifiers for the same training set, and then combine these weak classifiers to form a stronger final classifier.

LR^46^: LR is a nonlinear regression model. The feature data can be continuous or categorical variables. It can be used for classification, sorting and prediction problems.

RF^47^: Random forest is composed of many independent decision trees. It forms the final sample prediction by summarizing the prediction results of all decision trees. And the target result is obtained by voting or weighted average calculation of the predictions of all trees.

SVM^48^: It is a classic supervised learning algorithm used to solve binary and multi-classification problems. Its core idea is to classify data features by finding an optimal hyperplane in the feature space and try to maximize the interval of the hyperplane.

OnlineIDS^49^: It is an online intrusion detection system based on an adaptive random forest classifier and adaptive window drift detection, designed for real-time defense against cyberattacks in the charging infrastructure of electric vehicles.

PLE-MTL^50^: It is a novel multi-task learning architecture based on progressive layered extraction and hierarchical expert networks, enabling adaptive feature sharing for personalized recommendation scenarios.

### Experimental environment and parameters

All experiments with the EVSEMTLIDS model conduct on a GPU server equipped with a NVIDIA GeForce RTX 3090. The experimental running environment is shown in Table 4. The operating system is Windows 10 Professional, the GPU is a 3090 with 32GB of video memory, the CPU is an 11th Gen Intel(R) Core(TM) i5-1135G7 @ 2.40 GHz, and the RAM size is 64 GB (Table 5).

**Table 4:** The EVSEMTLIDS model experiment operating environment.

Number	Category	Size
1	Operating system	Windows 10
2	GPU	3090-32G
3	CPU	i5-1135
4	RAM	64G
5	Cuda	11.0
6	Python	3.7.0
7	torch	1.7.0

**Table 5:** The EVSEMTLIDS model of parameters.

Number	Category	Size
1	Hidden unit	32
2	Optimizer	Adam
3	Learning rate	0.001
4	Vector dimensions	128
5	Batch size	32
6	Drop	0.5
7	Parameter b	0.75
8	Epoch	50

The EVSEMTLIDS model builds a deep learning network intrusion detection architecture, which includes the following model parameters: the hidden unit size is 32, and the Adam optimizer is used to optimize the model. In addition, the model sets the learning rate to 0.001, the test data distribution ratio to 0.25, the representation vector dimension to 128, the training batch size to 32, the Epoch parameter to 50, the drop rate to 0.5, and the sparse feature classification ratio to 0.75. The parameters involved in the EVSEMTLIDS model are shown in the Table 4.

**Table 6:** The overall preformence of the EVSEMTLIDS model with the deep learning.

Task	Model	Metric			
		Acc	Pre	Recall	F1
Task1	DNN	0.9164	0.8995	0.8854	0.8921
	BPNN	0.9933	0.9885	0.9949	0.9917
	LSTM-Attention	0.9912	0.9830	0.9762	0.9780
	PLE-MTL	0.9939	0.9880	0.9949	0.9930
	**EVSEMTLIDS**	**0.9981**	**0.9965**	**0.9987**	**0.9976**
Task2	DNN	0.7581	0.8738	0.6926	0.6815
	BPNN	0.9844	0.9771	0.9776	0.9771
	LSTM-Attention	0.9831	0.9742	0.9655	0.9651
	PLE-MTL	0.9865	0.9798	0.9816	0.9782
	**EVSEMTLIDS**	**0.9957**	**0.9958**	**0.9935**	**0.9946**
Task3	DNN	0.6967	0.1299	0.1468	0.1327
	BPNN	0.7534	0.1238	0.1577	0.1378
	LSTM-Attention	0.7648	0.2017	0.1972	0.1843
	PLE-MTL	0.7679	0.2247	0.2106	0.1996
	**EVSEMTLIDS**	**0.9032**	**0.7368**	**0.6976**	**0.7013**

Significant values are in bold.

## Evaluation and discussion

### Overall performance

For RQ1, we compare the overall performance of the EVSEMTLIDS model in EVSE intrusion detection. Combining two types of detection models, deep learning and classic machine learning, the experiment verifies the effectiveness of the model through four types of detection indicators such as Acc and F1. First, through Table 6, we can find that the detection effect of the deep learning model is significantly weaker than that of the EVSEMTLIDS model in three different detection tasks. Among them, the experimental results of the DNN model are the worst. In Task 2, our model can improve the Acc metric by 23.76%, and the F1 metric in Task 2 can be improved by more than 31%. The performance of the BPNN model is similar to that of the LSTM-Attention. Its effect in the three detection tasks is better than that of the DNN model, but the experimental results are still weaker than the EVSEMTLIDS model. In the most complex classification task 3, the experimental effect of our model is the most outstanding, and it is significantly better than the other deep learning models in both Acc and F1 metrics.

The results in Table 6 show that while the PLE-MTL model performs well in feature decoupling, our EVSEMTLIDS model achieves superior performance in hierarchical consistency and error propagation mitigation, outperforming the PLE model in all three experimental tasks. Specifically, in Task 3, the PTM model improves Acc by 13.53% compared to the PLE model and by 50.17% in the F1 score. I believe this is mainly due to our model utilizing probabilistic priors and applying logical constraints to fine-grained predictions based on binary detection confidence. These findings empirically validate that the EVSEMTLIDS model is more effective in security domains with clear task hierarchies.

In addition, we also compared it with the classic machine learning model, and the experimental results are shown in Table 7. As can be seen from the table, compared with the relevant traditional machine learning models, the EVSEMTLIDS model still has better results in indicators such as Acc and F1. Compared with the experimental results of Task 1 and Task 2, the prediction performance of the traditional machine learning model has declined significantly. For example, the Acc metric of each model has dropped by more than 10%. However, the EVSEMTLIDS model has basically not changed much, which shows that our model is more stable and effective.

**Table 7:** The overall preformence of the EVSEMTLIDS model with the machine learning.

Task	Model	Metric			
		Acc	F1	Pre	Recall
Task1	Decision tree	0.9015	0.9019	0.9023	0.9015
	KNN	0.9134	0.9136	0.9138	0.9134
	Adaboost	0.9063	0.9045	0.9049	0.9064
	Naive Bayes	0.7980	0.8078	0.8461	0.7981
	LR	0.7219	0.7371	0.8313	0.7219
	Random forest	0.9107	0.9106	0.9105	0.9107
	SVM	0.8336	0.8339	0.8341	0.8336
	OnlineIDS	0.9913	0.9956	**0.9999**	0.9914
	**EVSEMTLIDS**	**0.9981**	**0.9965**	0.9987	**0.9953**
Task2	Decision Tree	0.7492	0.7500	0.7508	0.7492
	KNN	0.7854	0.7773	0.7739	0.7854
	Adaboost	0.5832	0.5695	0.5689	0.5832
	Naive Bayes	0.6549	0.6082	0.5733	0.6549
	LR	0.6209	0.5730	0.5372	0.6209
	Random forest	0.7887	0.7822	0.7783	0.7887
	SVM	0.7692	0.7140	0.8192	0.7692
	OnlineIDS	0.9840	0.9831	0.9840	0.9840
	**EVSEMTLIDS**	**0.9957**	**0.9946**	**0.9958**	**0.9935**

Significant values are in bold.

Secondly, Table 7 shows that our model surpasses the online IDS model in two of the four binary classification metrics. In multi-classification, our model surpasses the onlineIDS model in all four metrics, with improvements of 0.68% in Acc and 0.09% in F1. Therefore, our method outperforms the online IDS model in seven of the eight comparisons. In addition, compared to other machine learning models, the results of EVSEMTLIDS model in task 1 are significantly improved. We can found the results in Table 7. Among them, compared with the LR model, our model has improved the most, EVSEMTLIDS model Recall value has increased by more than 27.34%, and the F1 value has increased by 25.94%. Compared with the KNN model which is the best performing machine learning model, the detection performance of the EVSEMTLIDS model is also better. In terms of accuracy, recall, precision, and F1 score, the EVSEMTLIDS model outperformed the KNN model by more than 24% in Task 2.

### Analysis of performance of EVSEMTLIDS

**Fig. 5:**
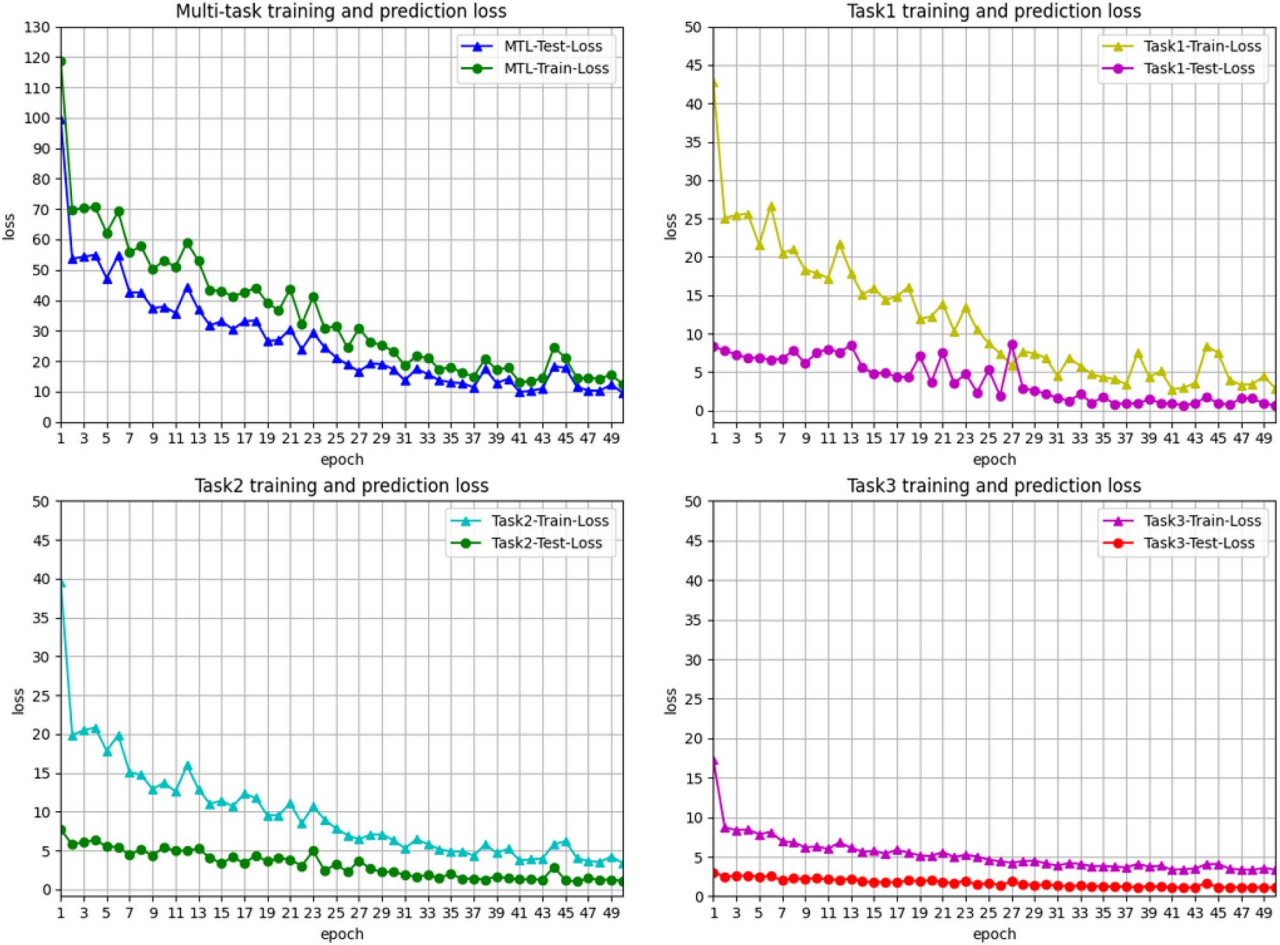
The Loss value curve of EVSEMTLIDS model and performance of each step.

For RQ2, in order to verify the effectiveness of the model. We calculated the training and loss function values of each detection task and plotted the loss curves of the multi-task as a whole and each task, so as to understand the overall change trend of the model for each task. As can be seen from Fig. 5, there are a total of four sub-graphs. Among them, the loss curve of the first multi-task graph is obtained by adding the loss values of each task. From the figure, it can be seen that the curves of the training and loss functions show a clear downward trend. However, when $Epoch=44$, there is a significant fluctuation in the loss value, which is mainly due to the change in the loss values of Task 1 and Task 2 that affects the overall loss value. In addition, the loss value curves of Task 1 and Task 2 are basically consistent with the structure of the multi-task loss value graph. The decline in both is large in the early stage and relatively stable in the later stage. In particular, the training loss curves of Task 1 and Task 2 basically stabilize after $Epoch=29$. For Task 2, due to its influence through the parameter transfer mechanism, the loss value of Task 2 can decrease faster and maintain good stability. We can see from Fig. 5 that after $Epoch>20$, the change in the test loss value is no longer obvious and gradually stabilizes.

**Fig. 6:**
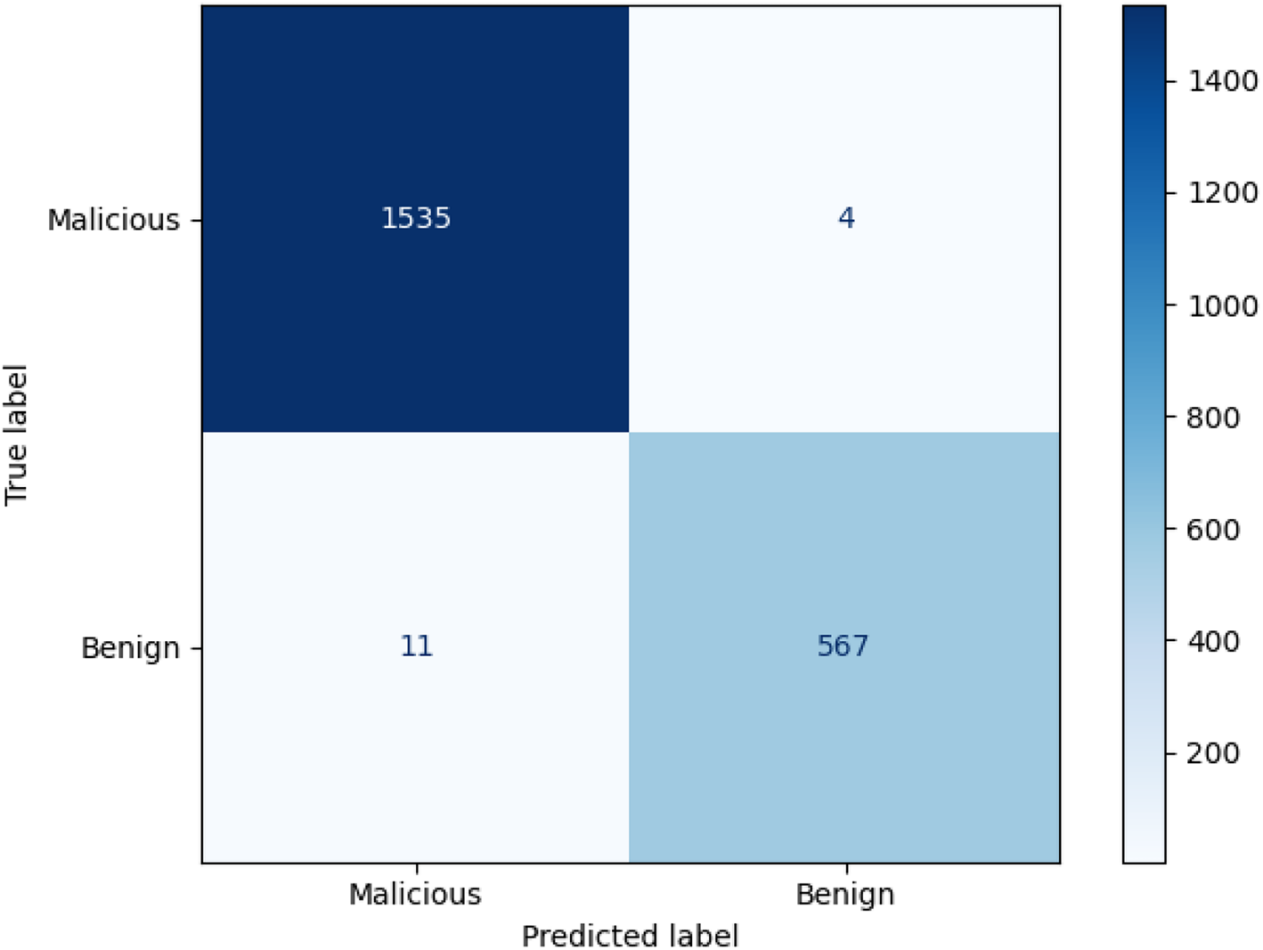
Task 1 experiment results.

**Fig. 7:**
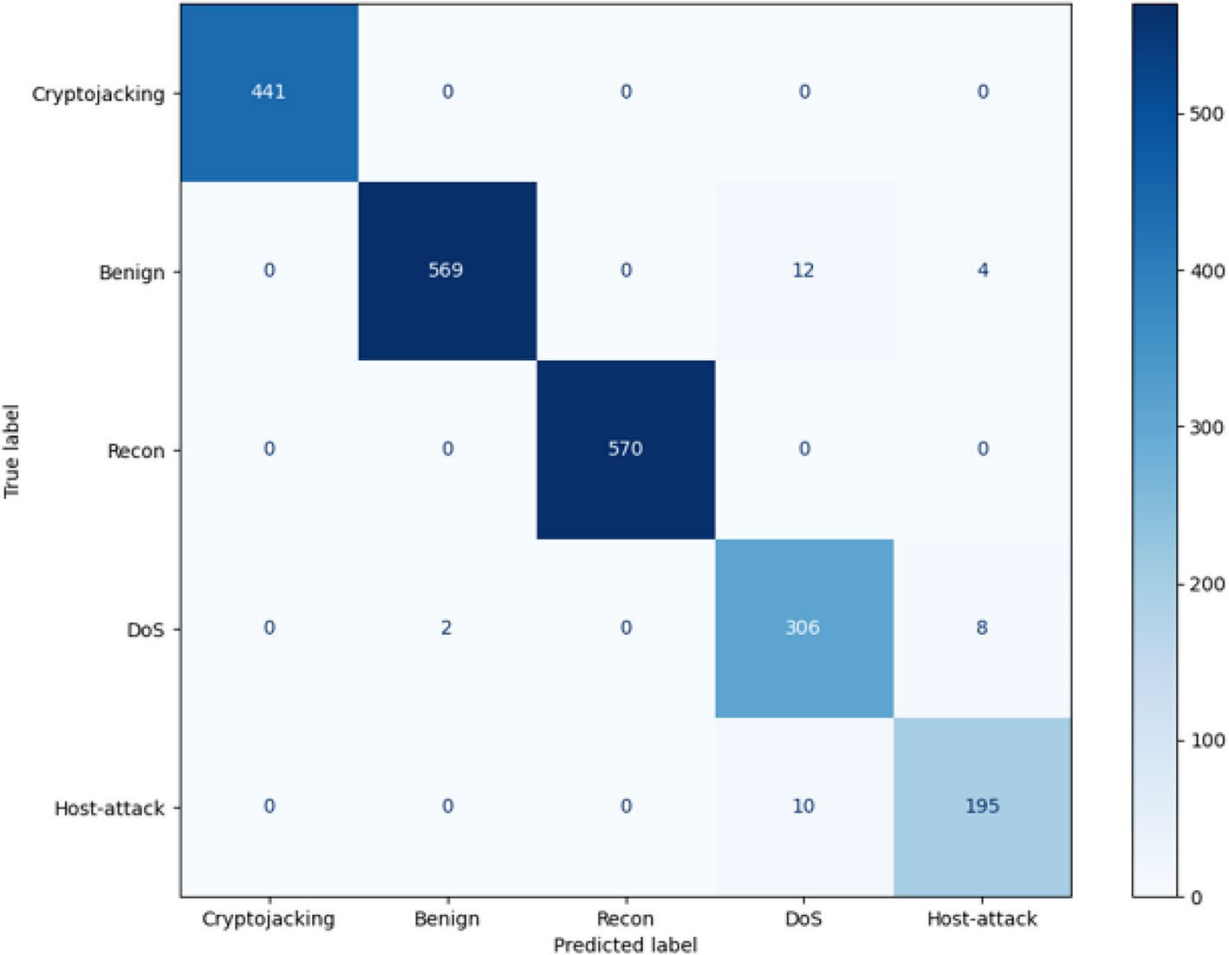
Task 2 experiment results.

For Task 3, the change is the smallest. This is because we used the probability transfer mechanism. The main classification results have been determined in Tasks 1 and 2, so the loss value in Task 3 did not fluctuate much and quickly stabilized. This shows that the probability transfer mechanism can effectively transfer the prediction probabilities of each task, speeding up the model fitting efficiency. In addition, in the later training process, we increased the range of Epochs and found that the overall loss value tended to be more stable. In addition, we verify the experimental results of each task by drawing classification diagrams of the three task categories. As shown in Figs. 6 and 7, the false alarm rate of malicious and benign samples in Task 1 is low, and they can basically be accurately predicted. In Task 2, the labels of the data are further refined, and the prediction effect is also more obvious. The two types of malicious data, portscan and cryptojacking, can still achieve 100% prediction accuracy.

**Fig. 8:**
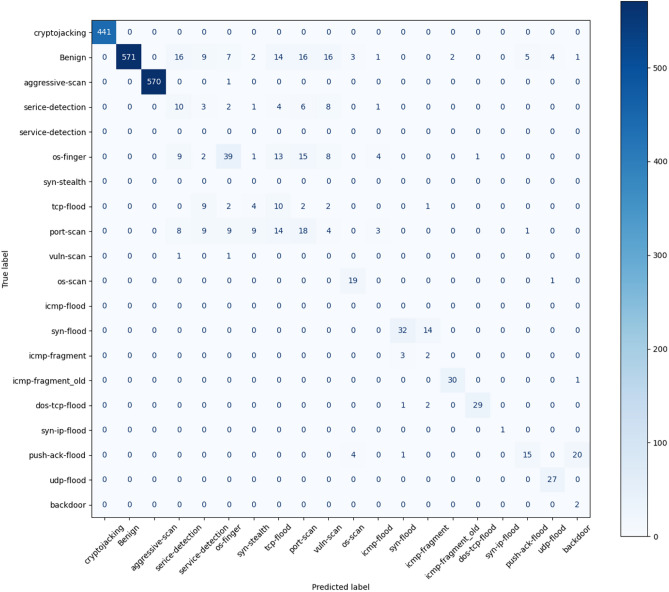
Task 3 experiment results.

Due to the large number of categories in Task 3, the number of samples of some data is small, resulting in poor detection effect of the model. As a result, the overall prediction accuracy of the model is low. However, as can be seen from Fig. 8, the EVSEMTLIDS model still maintains a high prediction accuracy for categories with a large amount of data.

### Parameters discussion

**Fig. 9:**
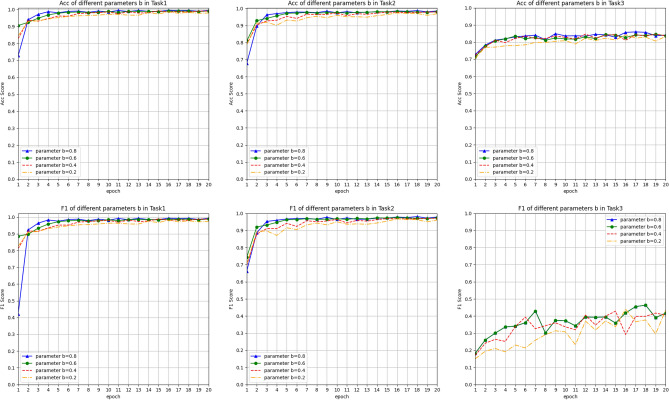
Performance sensitivity analysis with respect to parameters *a* and *b*.

For RQ3, we conducted an experimental analysis on the sparse feature classification parameter b. We verified its impact on the EVSEMTLIDS model by adjusting different parameter b. And for the three classification tasks, we analyzed the impact of the parameters on the model by drawing curves of two important indicators of the model, Acc and F1. From Fig. 9, we can see that in Task 1 and Task 2, when parameter $b=0.8$, the blue line of Acc and F1 converges faster than the other three lines, and the predicted value is also higher. This phenomenon shows that a larger classification parameter b can effectively improve the model data representation of sparse features, thereby improving the model’s detection effect. However, in Task 3, the experimental results of parameters $b=0.8$ and $b=0.6$ are not much different, which shows that in more category prediction tasks, a larger classification parameter b cannot further improve the classification effect of the model.

### Ablation experiment

For RQ4, we use component ablation to verify the impact of each key component on the detection model. The experiment constructed multiple detection models without multi-task learning architecture, probability transfer, and parameter adjustment. In addition, the experiment used 5 classification tasks as experimental targets to analyze the experimental effects of the detection model.

**Table 8:** Ablation experiment result.

Model	Metric		
	Accuracy	Recall	F1
W/O MTL	0.7874	0.7683	0.7264
W/O PT	0.9074	0.9183	0.8964
W/O Parameter	0.9467	0.9367	0.9388
**EVSEMTLIDS**	**0.9915**	**0.9887**	**0.9908**

Significant values are in bold.

As can be seen from the Table 8, the model without multi-task learning architecture has the worst detection effect, which shows that the feature sharing mechanism of the multi-task learning framework can not only improve the detection efficiency of the model, but also effectively improve the detection accuracy of the model. In addition, the probability transfer mechanism also has a great impact on the performance of the detection model. The detection effect of the model without the probability transfer mechanism also decreases significantly. The main reason for this is that the probability transfer mechanism can increase the correlation between the multi-task learning framework, so that the experimental effects of each prediction task of the model are effectively improved. Finally, the control parameters of the lack of sparse and dense features have the least impact on the model. The experimental results show that if the features are randomly divided, the prediction accuracy will be reduced by about 4.5%.

### Time consumption

For RQ5, we compared the training time of multiple detection models. Since the traditional machine learning models, DNN and BPNN deep learning models have relatively simple structures and their detection effects are very poor, we did not compare these models, but used the LSTM-Attention model with the best effect and more complex structure to compare the time consumption. In addition, we also considered not using the multi-task learning framework model for time consumption comparison to verify the effectiveness of the multi-task model. From the table 9, we can see that the time consumption of the EVSEMTLIDS model for a single epoch is only 14.5 seconds, and the 50-round model training takes a total of 725 seconds.

**Table 9:** Time consumption.

Model	Single epoch	50 epochs	Each instance
**EVSEMTLIDS**	**14.5s**	**725s**	**0.0021s**
W/T MTL	22.75s	1136s	0.0034s
LSTM-Attention	31.42s	1157	0.0046s
OnlineIDS	25.06s	1253	0.0037s

Significant values are in bold.

Moreover, without considering the multi-task framework, the model takes 22.75 seconds to train the detection model for each epoch, and 1136 seconds for 50 rounds of model training, which increases the overall experimental time by 56.69%. Combined with the experimental results in Table 9, it can be seen that the model can not only reduce the detection experiment, but also reduce the detection time. Finally, we found that the LSTM-Attention model has the longest detection time, with each epoch consuming 31.42 seconds, which is 116% higher than the EVSEMTLIDS model. In addition, the detection time of each sample of onlineIDS is 0.0037s, while the detection time of a single sample of our model is 0.0021s, so the detection time of a single sample is shortened by 0.0016s, and the overall time is reduced by about 43.24%. In general, the use of a multi-task learning model can effectively reduce the detection time.

Moreover, without considering the multi-task framework, the model takes 22.75 seconds to train the detection model for each epoch, and 1136 seconds for 50 rounds of model training, which increases the overall experimental time by 56.69%. Combined with the experimental results in Table 9, it can be seen that the model can not only reduce the detection experiment, but also reduce the detection time. Finally, we found that the LSTM-Attention model has the longest detection time, with each epoch consuming 31.42 seconds, which is 116% higher than the EVSEMTLIDS model. In addition, the detection time of each sample of onlineIDS is 0.0037s, while the detection time of a single sample of our model is 0.0021s, so the detection time of a single sample is shortened by 0.0016s, and the overall time is reduced by about 43.24%. In general, the use of a multi-task learning model can effectively reduce the detection time.

To evaluate the practical feasibility of EVSEMTLIDS, we measured its resource consumption on a computing platform with a 3090 GPU. Experimental results show that when processing a high-capacity dataset containing 8468 samples, the model consumes approximately 1.026 GB of dedicated process memory, with each sample requiring only 0.1241 MB of memory, and consumes 25.6% of CPU resources. These metrics demonstrate high throughput and low single-sample latency, confirming that the architecture is lightweight enough to be deployed on modern electric vehicle charging equipment edge controllers. Notably, the local execution of the DNN+CrossNet pipeline eliminates the need for continuous high-bandwidth data transmission to a central analyzer, thus meeting the real-time detection constraints and communication efficiency requirements of large-scale distributed charging networks.

### Model generalization

For RQ6, to further validate the generalization ability and robustness of the EVSEMTLIDS framework, we conducted extensive comparative experiments using the benchmark datasets CICIDS2017 and CIC-UNSW-NB15. Performance benchmarks focused on the best-performing architectures among the two benchmark models: OnlineIDS and PLE-MTL. To address the class imbalance prevalent in network intrusion detection datasets—where benign traffic typically dominates—we preprocessed the CICIDS2017 and CIC-UNSW-NB15 datasets by removing excessive benign samples, ensuring the total counts of benign and malicious traffic were equalized. This step creates a balanced training environment, enabling MTL models to effectively learn patterns from both normal and malicious activities without bias toward the majority class.

For the CICIDS2017 dataset (Table 10), after balancing, both benign and malicious samples total 425,694 in Task 1, a binary classification task distinguishing normal from malicious traffic. In Task 2, which focuses on multi-class attack detection, the dataset retains 425,694 benign samples and includes diverse attack categories: DoS (193,745), DDoS (128,014), Port Scanning (90,694), Brute Force (9,150), Web Attacks (2,143), and Bots (1,948).

**Table 10:** CICIDS2017 dataset statistics.

Task1		Task2			
Category	Total	Category	Total	Category	Total
Benign	425694	Benign	425694	DDoS	128014
Malicious	425694	DoS	193745	Port scanning	90694
		Brute force	9150	Web attacks	2143
		Bots	1948		

For the CIC-UNSW-NB15 dataset (Table 11), the balanced Task 1 contains 89,583 benign and 89,583 malicious samples, providing a comparable binary classification task. In Task 2, the dataset maintains 89,583 benign samples and encompasses a broader spectrum of attack types: Exploits (30,951), Fuzzers (29,613), Reconnaissance (16,735), Generic (4,632), DoS (4,467), Shellcode (2,102), Analysis (385), Backdoor (452), and Worms (246).

**Table 11:** CIC-UNSW-NB15 dataset statistics.

Task1		Task2			
Category	Total	Category	Total	Category	Total
Benign	89583	Benign	89583	Reconnaissance	16735
Malicious	89583	Generic	4632	Shellcode	2102
		Exploits	30951	Fuzzers	29613
		DoS	4467	Backdoor	452
		Worms	246	Analysis	385

On the CICIDS2017 dataset, our proposed EVSEMTLIDS model demonstrates superior performance across both tasks compared to the OnlineIDS and PLE-MTL baselines, as shown in Table 12. For Task 1 , EVSEMTLIDS achieves an accuracy of 0.9499, precision of 0.9502, recall of 0.9499, and F1-score of 0.9499, outperforming OnlineIDS and PLE-MTL by a clear margin. In Task 2, multi-class attack detection, while all models experience a performance drop due to increased task complexity, EVSEMTLIDS still maintains the highest scores: accuracy of 0.9604, precision of 0.7489, recall of 0.6666, and F1-score of 0.6776, significantly exceeding OnlineIDS and PLE-MTL. These results validate the effectiveness of our multi-task learning framework in handling both binary and multi-class intrusion detection challenges on CICIDS2017.

**Table 12:** Overall performance comparison of the EVSEMTLIDS model with baselines on the CICIDS2017 dataset.

Task	Model	Metric			
		Acc	Pre	Recall	F1
Task1	OnlineIDS	0.9287	0.9309	0.9376	0.9321
	PLE-MTL	0.9106	0.9146	0.9203	0.9154
	**EVSEMTLIDS**	**0.9499**	**0.9502**	**0.9499**	**0.9499**
Task2	OnlineIDS	0.8973	0.6355	0.5449	0.5954
	PLE-MTL	0.8654	0.6043	0.5342	0.5461
	**EVSEMTLIDS**	**0.9604**	**0.7489**	**0.6666**	**0.6776**

Significant values are in bold.

On the CIC-UNSW-NB15 dataset, EVSEMTLIDS consistently outperforms the baseline models, and Table 13 showcases its robustness across diverse attack scenarios. In Task 1, EVSEMTLIDS achieves exceptional results with an accuracy of 0.9846, precision of 0.9849, recall of 0.9849, and F1-score of 0.9846, surpassing OnlineIDS and PLE-MTL. For Task 2, which involves classifying a wider range of attack types, EVSEMTLIDS again delivers the best performance with an accuracy of 0.8326, precision of 0.5722, recall of 0.4046, and F1-score of 0.4007, outperforming OnlineIDS and PLE-MTL. The consistent superiority of EVSEMTLIDS on both datasets confirms its ability to generalize well and effectively handle the complexities of network intrusion detection in real-world scenarios.

**Table 13:** Overall performance comparison of the EVSEMTLIDS model with baselines on the CIC-UNSW-NB15 dataset.

Task	Model	Metric			
		Acc	Pre	Recall	F1
Task1	OnlineIDS	0.9642	0.9592	0.9496	0.9537
	PLE-MTL	0.9539	0.9308	0.9046	0.9276
	**EVSEMTLIDS**	**0.9846**	**0.9849**	**0.9849**	**0.9846**
Task2	OnlineIDS	0.7741	0.5208	0.3627	0.3546
	PLE-MTL	0.7279	0.4836	0.3296	0.3284
	**EVSEMTLIDS**	**0.8326**	**0.5722**	**0.4046**	**0.4007**

Significant values are in bold.

Overall, our model performs well on all three datasets. To facilitate the reproduction of our results, we have released the code and output of our model. Due to the large size of the dataset, we packaged the code and dataset together and uploaded them to Google Drive (https://drive.google.com/file/d/1oDEagXda-3DvqDoYCrFaXOyy4v7-irAq/view?usp=drive_link).

## Conclusion and future works

In this paper, we aim to solve the problems of high overlap of traffic features and low recognition of detection models in traditional EVSE intrusion detection. We propose a deep learning detection model EVSEMTLIDS based on a multi-task learning architecture to deeply analyze the malicious intrusion behavior of EVSE. First, in order to avoid the impact of high overlap of EVSE traffic features on the performance of the detection model, we use the host features of the EVSE to study the intrusion results. Subsequently, in order to improve the representation effect of the detection model data, this paper combines DNN and CrossNet deep neural networks to represent the host data. Experimental results show that the EVSEMTLIDS model we proposed can effectively detect intrusion events and reduce the detection time. At the same time, the probability migration mechanism we proposed can improve the prediction performance between multiple tasks.

Despite these advantages, the EVSEMTLIDS model still has limitations. For example, the choice of hyperparameters, such as the learning rate, is crucial to the model’s success and requires careful tuning. Furthermore, the model’s feature interpretability is insufficiently explored, making it difficult to identify intrusion paths.

In the future, we will focus on studying the impact of EVSE intrusion on the entire power grid, thereby effectively curbing its impact. Furthermore, we will incorporate online learning or incremental learning methods to enhance its scalability.

## Data Availability

The datasets used and analysed during the current study are available from the corresponding author on reasonable request.
